# Edible Bird's Nest Prevents High Fat Diet-Induced Insulin Resistance in Rats

**DOI:** 10.1155/2015/760535

**Published:** 2015-07-27

**Authors:** Zhang Yida, Mustapha Umar Imam, Maznah Ismail, Der-Jiun Ooi, Nadarajan Sarega, Nur Hanisah Azmi, Norsharina Ismail, Kim Wei Chan, Zhiping Hou, Norhayati Binti Yusuf

**Affiliations:** ^1^Laboratory of Molecular Biomedicine, Institute of Bioscience, Universiti Putra Malaysia, 43400 Serdang, Selangor, Malaysia; ^2^Cardiology Department, Affiliated Hospital of Chengde Medical University, Chengde, Hebei 067000, China; ^3^Department of Nutrition and Dietetics, Faculty of Medicine and Health Sciences, Universiti Putra Malaysia, 43400 Serdang, Selangor, Malaysia

## Abstract

Edible bird's nest (EBN) is used traditionally in many parts of Asia to improve wellbeing, but there are limited studies on its efficacy. We explored the potential use of EBN for prevention of high fat diet- (HFD-) induced insulin resistance in rats. HFD was given to rats with or without simvastatin or EBN for 12 weeks. During the intervention period, weight measurements were recorded weekly. Blood samples were collected at the end of the intervention and oral glucose tolerance test conducted, after which the rats were sacrificed and their liver and adipose tissues collected for further studies. Serum adiponectin, leptin, F2-isoprostane, insulin, and lipid profile were estimated, and homeostatic model assessment of insulin resistance computed. Effects of the different interventions on transcriptional regulation of insulin signaling genes were also evaluated. The results showed that HFD worsened metabolic indices and induced insulin resistance partly through transcriptional regulation of the insulin signaling genes. Additionally, simvastatin was able to prevent hypercholesterolemia but promoted insulin resistance similar to HFD. EBN, on the other hand, prevented the worsening of metabolic indices and transcriptional changes in insulin signaling genes due to HFD. The results suggest that EBN may be used as functional food to prevent insulin resistance.

## 1. Introduction

The growing burden of cardiometabolic diseases, even in the face of increasing advances in medical sciences, is the driving factor behind the heightened interest in alternative therapies in the management of these diseases and associated problems [1, 2]. Additionally, rising obesity rates globally due to unhealthy lifestyle factors promote these rising disease trends; obesity promotes insulin resistance and eventually cardiometabolic diseases [3]. In fact, it is estimated that if persons at risk of insulin resistance and cardiometabolic diseases are accurately determined using sensitive diagnostic techniques, the numbers of those needing interventions to manage their conditions would be much higher than established figures [4]. There are different theories used to hypothesize the underlying mechanisms involved in the progression from obesity to insulin resistance and cardiometabolic diseases. Popularly, excess calories are thought to promote deposition of visceral fat around organs, with consequent changes in the adipose tissue metabolism in the body, and ultimately increase in insulin resistance especially in liver, as a result of glucolipotoxicity [5]. The ensuing insulin resistance causes disruption in the propagation of insulin signals on insulin-responsive cells. In fact, the perceived role of this phenomenon is the reason why therapeutic approaches to the management of insulin resistance and other associated cardiometabolic diseases involve the use of agents that promote insulin signaling.

Edible bird's nest (EBN) is traditionally consumed among Asians for its nutritional value. It is believed to enhance energy levels, prevent aging, and improve overall well-being. Furthermore, there are scientific reports of its antioxidative, anti-inflammatory, and bone-strengthening effects [6–9]. However, its effects on insulin resistance and cardiometabolic indices have not been documented. In view of the large patronage of EBN by Asians, especially of Chinese origin [10], we decided to evaluate the effects of EBN consumption on cardiometabolic indices in high fat diet- (HFD-) fed rats. Based on the anti-inflammatory and antioxidant effects of EBN, we assumed it would have favorable effects on cardiometabolic indices, since both effects have been reported to favor insulin sensitivity. As the first study of its kind, we hypothesized that the results could provide the evidence for continued use of EBN as a supplement and may even pave way for evidence-based development of functional foods and nutraceuticals using EBN for managing cardiometabolic diseases.

## 2. Materials and Methods

### 2.1. Materials

Leptin, F2-isoprostane, and insulin ELISA kits were purchased from Elabscience Biotechnology Co., Ltd (Wuhan, China), while adiponectin ELISA kit was from Millipore (Billerica, MA, USA). Lipid profile kits were purchased from Randox Laboratories Ltd (Crumlin, County Antrim, UK). GenomeLab GeXP Start Kit was from Beckman Coulter Inc (Miami, FL, USA), and RNA extraction kit was from RBC Bioscience Corp. (Taipei, Taiwan). Simvastatin was from Pfizer (New York, NY, USA) and RCL2 Solution from Alphelys (Toulouse, France). Analytical grade ethanol was purchased from Merck (Darmstadt, Germany). Cholesterol and cholic acid were purchased from Amresco (Solon, OH, USA) and Santa Cruz Biotechnology (Santa Cruz, CA, USA), respectively. Standard rat pellet was from Specialty feeds (Glen Forrest, WA, USA), while palm oil was supplied by Yee Lee Edible oils Sdn. Bhd. (Perak, Malaysia). EBN, of* Aerodramus fuciphagus* (white nest swiftlet) origin, supplied by Blossom View Sdn. Bhd (Terrengganu, Malaysia) was cleaned under tap water for 5 mins, dried at room temperature, and ground into powder manually using mortar and pestle before incorporating it into rat pellet.

### 2.2. Bioactive and Proximate Analyses

The proximate analysis of EBN was done as reported in our previous publication [11], based on the official methods of Association of Official Analytical Chemists. Briefly, nitrogen content was determined using micro-Kjeldahl apparatus (Kjeltech 2200 Auto Distillation Unit, FOSS Tecator, Hoganas, Sweden), and then protein content was determined as N × 5.95. Furthermore, the ashing process was done by incinerating the sample in a furnace (Furnace 62700, Barnstead/Thermolyne, Dubuque, IA, USA) set at 550 C, while the fat content was determined as the dried ether extract of EBN. Then, carbohydrate content was determined using the following formula: (100% – protein content – moisture content – ash content – crude fat content). All results were expressed as percentage of dry weight. The amounts of major bioactives in EBN (sialic acid [SA], lactoferrin [LF], and ovotransferrin [OVF]) were analyzed using ELISA-based techniques (LF and OVF) and HPLC-DAD (SA). Briefly, EBN was ground to powder and dissolved in water at 37°C for 2 h on a shaking incubator (LSI-3016, Daihan Lab tech Co. Ltd, Korea) and finally filtered. The water extract was then used to detect LF and OVF concentrations using Chicken Lactoferrin and Ovotransferrin Elisa Kits, Biosource (San Diego, California, USA), according to manufacturer's instructions. Additionally, water extract of EBN was also analysed for SA content using HPLC-DAD as reported previously [12].

### 2.3. Animal Study

The Animal Care and Use Committee (ACUC) of the Faculty of Medicine and Health Sciences, Universiti Putra Malaysia, approved the use of animals in this study (Project approval number UPM/IACUC/AUP-R011/2014), and animals were handled as stipulated by the guidelines for the use of animals. Sprague Dawley rats (10-week old, 230–280 g, *n* = 30) were housed at the animal house (25 ± 2°C, 12/12 h light/dark cycle) and allowed to acclimatize for 2 weeks with free access to normal pellet and water. After acclimatization, rats were fed HFD containing 4.5% cholesterol and 0.5% cholic acid with or without treatment using simvastatin or EBN (Table 1), except the normal group (*n* = 6). Intervention lasted for another 12 weeks, after which rats were sacrificed and their organs harvested for further studies. Additionally, blood samples were collected at the end of the intervention for biochemical analyses.

### 2.4. Food Intake and Weight

Food intake was calculated by subtracting the leftover food from what was added the previous day. Weight was recorded after acclimatization and weekly thereafter until sacrifice.

### 2.5. Biochemical Analyses

Lipid profile analyses were performed using serum from blood collected at the beginning and end of the study by cardiac puncture after an overnight fast. Samples were analyzed using Randox analytical kits according to manufacturer's instructions using a Selectra XL instrument (Vita Scientific, Dieren, The Netherlands). Blood glucose was measured using glucometer (Roche Diagnostics, Indianapolis, IN, USA), and homeostatic model assessment of insulin resistance (HOMA-IR), a measure of insulin sensitivity, was computed from the fasting plasma glucose and insulin levels using the formula, HOMA-IR = (fasting glucose level [mg/dL]/fasting plasma insulin [uU/mL])/2430 [13].

### 2.6. Serum Adiponectin, Leptin, F2-Isoprostane, and Insulin

Serum from blood collected in plain tubes was used for measurements of adiponectin, leptin, F2-isoprostane, and insulin using the respective ELISA kits according to the manufacturers' instructions. Absorbance was read on BioTeK Synergy H1 Hybrid Reader (BioTek Instruments Inc., Winooski, VT, USA) at the appropriate wavelengths (450 nm for insulin, leptin, and F2-isoproatane and 450 and 590 for adiponectin). The results were analyzed on http://www.myassays.com/ using four parametric test curve: adiponectin (*R* ^2^ = 0.9914), insulin (*R* ^2^ = 1), leptin (*R* ^2^ = 0.9996), and F2-isoprostane (*R* ^2^ = 1).

### 2.7. Gene Expression

#### 2.7.1. Primer Design

*Rattus norvegicus* gene sequences from the National Center for Biotechnology Information website (http://www.ncbi.nlm.nih.gov/nucleotide/) were used to design primers (Table 2) on GenomeLab eXpress Profiler software. In addition to the genes of interest, primers were also designed for housekeeping genes, while the internal control (Kanr) was supplied by Beckman Coulter Inc. Primers were tagged with an 18-nucleotide universal forward and 19-nucleotide universal reverse sequence, respectively. Primers were supplied by Integrated DNA Technologies (Singapore) and reconstituted in RNAse free water.

#### 2.7.2. RNA Extraction, Reverse Transcription, and PCR

RNA was extracted from liver and adipose tissues using the total RNA isolation kit (RBC Biotech Corp., Taipei, Taiwan) according to the manufacturer's instructions. Reverse transcription (20 ng) and PCR were done according to the GenomeLab GeXP Start Kit protocol (Beckman Coulter, USA), using the conditions shown in Table 3.

#### 2.7.3. GeXP Genetic Analysis System and Multiplex Data Analysis

**Figure 1:**
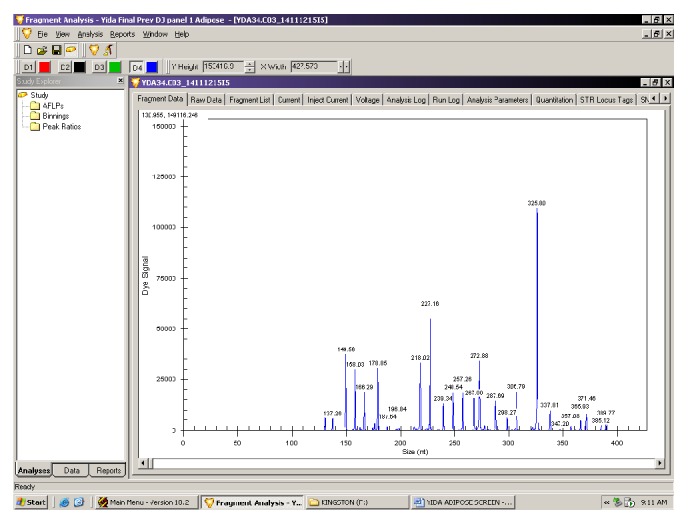
Representative electropherogram following gene expression analysis on GenomeLab GeXP genetic analysis system (Beckman Coulter Inc., USA). The genes and their expected sizes were Irs2-137; Slc2a2-149; Kcnj11-158; Insr-166; Glut4-178; Irs1-188; Gck-197; Mapk8-218; Pklr-227; Prkcd-239; B2m-248; Hprt1-257; Mapk1-268; Socs1-272; Rpl13a-287; Prkcz-298; Ikbkb-306; Kan(r)-325; Mtor-337; Pdx1-348; Pik3cd-357; Actb-365; Pik3r1-372; Pik3ca-385; Hk2-389.

PCR products (1 uL) were mixed with 38.5 *μ*L sample loading solution and 0.5 *μ*L DNA size standard 400 (GenomeLab GeXP Start Kit; Beckman Coulter, Inc, USA) on a 96-well sample plate and loaded on the GeXP genomelab genetic analysis system (Beckman Coulter, Inc, Miami, FL, USA), which separates PCR products based on size by capillary gel electrophoresis.  Figure 1 shows a representative electropherogram. Results were analyzed with the Fragment Analysis module of the GeXP system software and normalized on the eXpress Profiler software.

### 2.8. Data Analysis

The means ± standard deviations (*n* = 6) of the groups were used for the analyses. One-way analysis of variance (ANOVA) was performed using SPSS 17.0 software (SPSS Inc., Chicago, IL, USA) to assess the level of significance of differences between means with a cutoff of *P* < 0.05.

## 3. Results and Discussions

### 3.1. Proximate and Bioactive Analyses

The proximate analysis of EBN showed that it contained mostly protein and carbohydrates (Table 3), in agreement with previous findings [10]. Additionally, it contained a significant amount of SA (11%) as bioactive, with lesser amounts of LF (1%) and OVF (0.4%). Previous reports have indicated that EBN is bioactive-rich [10], and it is likely that food synergy plays role in its overall effects [14]. The presence of any one bioactive compound may not explain the bioactivity of EBN, but the concentration of the leading bioactive compounds like SA may have an influence to a great extent, albeit with the contribution of other bioactives. Moreover, SA, LF, and OVF have all been reported to have varying functional effects [15, 16], and their synergism may even produce better. This is similar to the concept of bioactive-rich fraction we have advocated for recently, in which a lead bioactive compound in an extract produces better bioactivity in the presence of other bioactive compounds [17]. Therefore, in view of recent advocacy for the study of foods but not their individual constituents as the functional unit of nutrition [18], we decided to study the bioactivity of EBN as a whole.

### 3.2. Weight Changes

**Figure 2:**
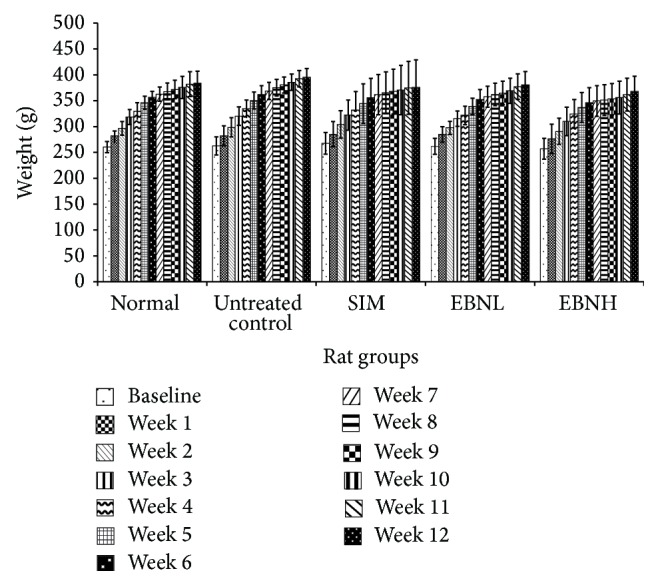
Effects of edible bird's nest (EBN) on body weight changes in high fat diet- (HFD-) fed rats over 12 weeks. The normal group received standard rat chow, while the other groups received HFD containing 4.5% cholesterol and 0.5% cholic acid (untreated control group), HFD containing 4.5% cholesterol and 0.5% cholic acid + 10 mg/kg/day simvastatin (SIM), HFD containing 4.5% cholesterol and 0.5% cholic acid + 2.5% EBN (EBNL, EBN low), or HFD containing 4.5% cholesterol and 0.5% cholic acid + 20% EBN (EBNH, EBN high).

Figure 2 shows the changes in body weights of rats over 12 weeks of intervention. No statistically significant changes were observed but the changes in HFD-fed (untreated control) group (50% increase) were higher, in comparison with normal (47%), simvastatin (40%), 2.5% EBN (45%), and 20% EBN (43%) groups. Interestingly, as shown in Table 3, calorie intake for the different groups was similar over the intervention period. The results indicated therefore that EBN had some weight-modulating properties, although the weight gain was lowest for simvastatin-treated group. Moreover, simvastatin is reported to have some weight reducing properties [19].

### 3.3. OGTT, Insulin, HOMA-IR, and Lipid Profile

Serum insulin levels at the end of intervention were not remarkably different between the groups except for the 2.5% EBN group, which was significantly lower (*P* < 0.05) than others (Table 4). However, absolute insulin levels may not reflect the state of the underlying insulin responsiveness since insulin resistance often starts with high insulin levels and ends up with lower levels. Therefore, we computed the HOMA-IR as a marker of insulin resistance that combines insulin levels and fasting glucose levels. The data showed that untreated control and simvastatin groups had a tendency to cause insulin resistance. This mirrors earlier findings on the effects of HFD feeding [20] and simvastatin [21] on development of insulin resistance. EBN groups had lower HOMA-IR values in comparison with other groups, although not significantly different from normal (both EBN groups) and untreated control (20% EBN group) groups.

**Figure 3:**
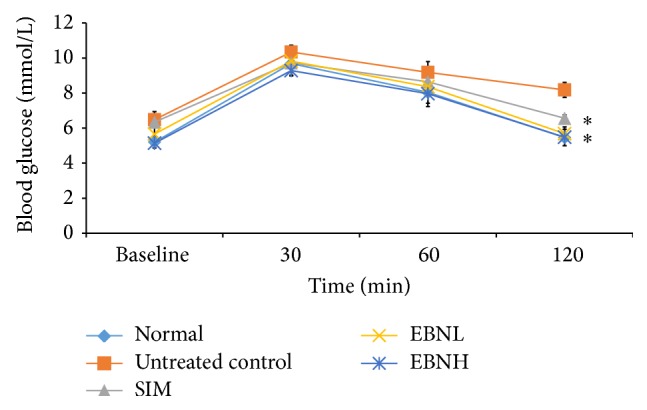
Effects of edible bird's nest (EBN) on oral glucose tolerance test in fed high fat diet- (HFD-) fed rats. Groupings are similar to Figure 2. ∗ indicates significant difference (*P* < 0.05) in comparison with untreated control.

The cholesterol levels in the untreated control group were significantly increased in comparison with the normal group (Table 4). Moreover, worsening of lipid profile has been associated with insulin resistance [22]. The total cholesterol was significantly reduced by simvastatin and 20% EBN group (*P* < 0.05). As seen from other cholesterol indices in the table, simvastatin, which is used to manage hypercholesterolaemia was able to improve lipid profile but not as well as 20% EBN treatment. Furthermore, Figure 3 shows the OGTT results for the intervention groups. The glycemic response for the diabetic untreated group was higher than other groups (*P* < 0.05), while the normal and EBN groups were the lowest and significantly lower than simvastatin treated group (*P* < 0.05). Insulin regulates a number of metabolic changes in the body and derangements in its actions even before insulin resistance becomes overt can be detected using the OGTT. This is because the OGTT gives an indication of how a biological system will respond in the presence of glucose and indicates how well the postglucose insulin surge handles the glycemic load received in the blood stream [23]. In this study, the data showed that untreated control and simvastatin groups did not handle the glucose load in a manner befitting the levels of insulin observed in the serum. Therefore, in spite of the lack of difference in insulin levels between the groups, the OGTT data showed that the untreated control and simvastatin-treated groups will have abnormal glycemic responses compared with the normal and EBN groups because their bodies were tending towards insulin resistance.

### 3.4. Serum Adiponectin, Leptin, and F2-Isoprostane

**Figure 4:**
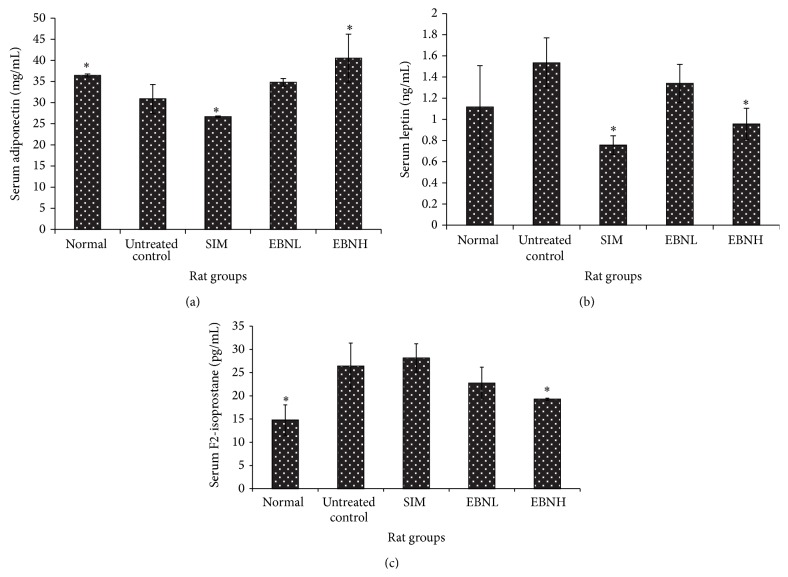
Effects of edible bird's nest (EBN) on (a) serum adiponectin, (b) serum leptin, and (c) serum F2-isoprostane in high fat diet- (HFD-) fed rats. Groupings are similar to Figure 2. ∗ indicates significant difference (*P* < 0.05) in comparison with untreated control.

Figure 4 shows the results for the serum levels of adiponectin, leptin, and F2-isoprostane. The results suggested worsened metabolic indices (increased leptin and F2-isoprostane and decreased adiponectin) in the untreated control group in comparison with the normal group. The EBN groups showed dose-dependent improvements (decreased leptin and F2-isoprostane and increased adiponectin) in the metabolic indices although only 20% EBN group was significantly better than the untreated control group. Adiponectin and leptin are adipokines that have an inverse relationship and have both been implicated in the development of insulin resistance. Low levels of adiponectin and high levels of leptin are indicative of a tendency for insulin resistance, while interventions that reverse these trends are reported to improve insulin sensitivity [24]. Furthermore, F2-isoprostane is a marker of oxidative stress, which is also linked with insulin resistance [25]. In fact, oxidative stress is hypothesized to precede insulin resistance [26], while antioxidants and interventions that lower oxidative stress levels are thought to improve insulin sensitivity [27]. Based on the trends observed in the present study, therefore, it can be argued that EBN prevented HFD-induced insulin resistance in rats, partly through its ability to reduce oxidative stress.

### 3.5. Hepatic and Adipose Tissue mRNA Levels of Insulin Signaling Genes

The data thus far indicated that EBN is able to prevent insulin resistance in rats fed HFD over 12 weeks. Additionally, the data showed that although simvastatin is able to produce lower levels of cholesterol, it, in fact, increases insulin resistance, in agreement with previous reports [21]. Based on the fact that insulin levels were similar between the groups in this study, but there were significant differences in insulin sensitivity, we hypothesized that changes in insulin sensitivity may have been mediated at insulin signaling level. We, therefore, determined the effects of our interventions on transcriptional regulation of insulin signaling genes (Table 2) in hepatic and adipose tissues.

**Figure 5:**
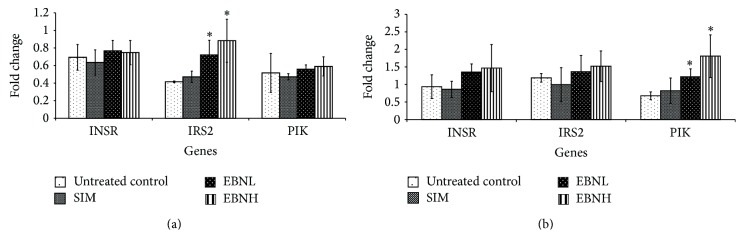
Effects of edible bird's nest (EBN) on (a) hepatic and (b) adipose tissue mRNA levels of insulin receptor (Insr), insulin receptor substrate (Irs) 2 and Phosphoinositide-3-kinase (PI3K) in high fat diet- (HFD-) fed rats. Groupings are similar to Figure 2. ∗ indicates significant difference (*P* < 0.05) in comparison with untreated control.

The expressions of the insulin signaling genes in hepatic and adipose tissues were characteristic of insulin resistance in the untreated control group; downregulation of the insulin receptor (Insr), insulin receptor substrate (IRS) 2, and phosphoinositide-3-kinase (PI3K) observed in the liver and adipose tissues in this group are suggestive of insulin resistance (Figure 5) [28–30]. Activation of Insr by insulin will normally initiate a cascade that involves activation of IRS and eventually PI3K, which mediate the intracellular actions of insulin. Transcriptional disruption of this insulin-initiated cascade forms part of the basis for obesity-induced insulin resistance [31].

**Figure 6:**
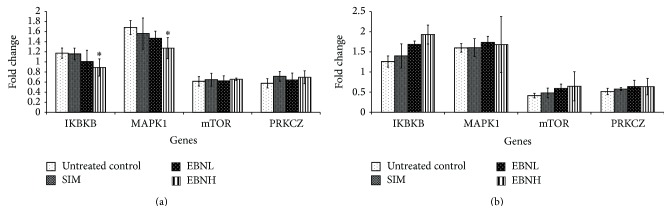
Effects of edible bird's nest (EBN) on (a) hepatic and (b) adipose tissue mRNA levels of mammalian target of rapamycin (mTOR), protein kinase C zeta (Prkcz), inhibitor of kappa light polypeptide gene enhancer in B-cells, kinase beta (IKBKB), and mitogen-activated protein kinase (MAPK) 1 in high fat diet- (HFD-) fed rats. Groupings are similar to Figure 2. ∗ indicates significant difference (*P* < 0.05) in comparison with untreated control.

Additionally, upregulation of mitogen-activated protein kinase (MAPK) [32] and inhibitor of kappa light polypeptide gene enhancer in B-cells, kinase beta (Ikbkb) [33] and downregulation of mammalian target of rapamycin (mTOR) [34] and protein kinase C, zeta (Prkcz) [35], as seen with the untreated control group (Figure 6) are thought to promote phosphorylation of IRS with consequent increase in insulin resistance due to disruption of IRS-mediated insulin action via activation of PI3K [28, 30]. Intervention with EBN upregulated the expression of Insr, IRS2 and PI3K in both liver and adipose tissues, but the difference was only significant for IRS2 in the liver and PI3K in the adipose tissue (Figure 5). These, however, suggest that EBN prevented HFD-induced insulin resistance through transcriptional regulation of insulin signaling genes. Moreover, EBN upregulated mTOR and Prkcz in the liver and adipose tissue but only caused downregulation of MAPK and Ikbkb in the liver indicating that the transcriptional changes induced by EBN had differential effects on insulin signaling genes in liver and adipose. Therefore, slightly different mechanisms may be involved in its enhanced insulin signaling in different tissues.

**Figure 7:**
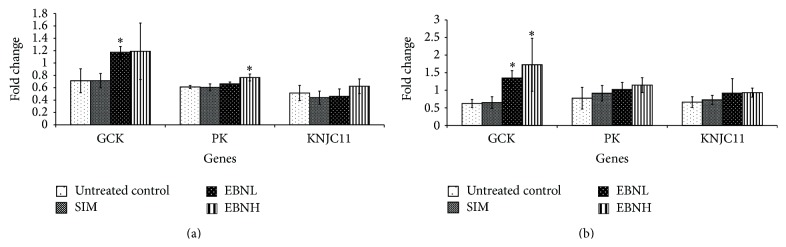
Effects of edible bird's nest (EBN) on (a) hepatic and (b) adipose tissue mRNA levels of Glucokinase (Gck), potassium inwardly rectifying channel, subfamily J, member 11 (KCNJ11), and pyruvate kinase-liver isoform (L-Pk) in high fat diet- (HFD-) fed rats. Groupings are similar to Figure 2. ∗ indicates significant difference (*P* < 0.05) in comparison with untreated control.

The activities of glucokinase (Gck) and pyruvate kinase (Pk) are affected in insulin resistance, decreasing the chances of intracellular glucose phosphorylation and its commitment to glycolysis [36]. In the adipose and liver tissues of untreated control group, we observed downregulation of the Gck and Pk genes, in line with increased insulin resistance (Figure 7). The levels of these genes are believed to directly influence the levels of cellular adenosine triphosphate (ATP) and consequently the activity of the potassium inwardly rectifying channel, subfamily J, member 11 (KCNJ11) gene, which regulates the ion channels involved in glucose sensing [37]. In this study, we observed downregulation of the KCNJ11 gene in both liver and adipose tissues, suggesting that the changes in Gck and Pk expression may have affected its expression through their effects on cellular ATP levels. EBN intervention was able to upregulate expressions of Gck, Pk, and KCNJ11 in both liver and adipose tissues.

**Figure 8:**
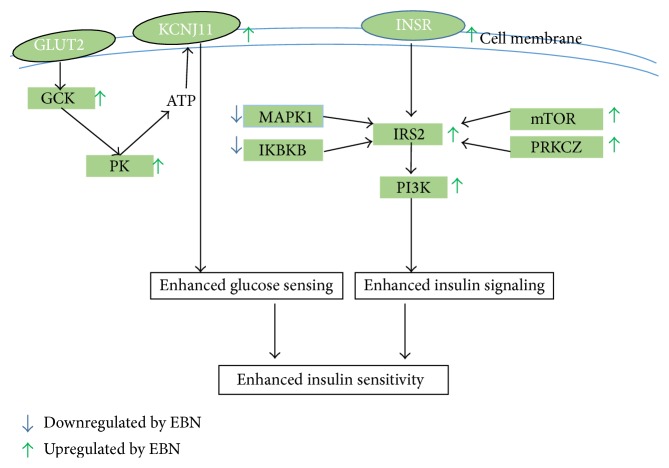
Proposed schematic showing targets of edible bird's nest (EBN) action in the insulin signaling pathway. EBN prevents insulin resistance in high fat diet rats by influencing the transcriptional regulation of multiple genes.

Based on the patterns of expression in the liver and adipose tissues, we propose that EBN may be exerting its effect on insulin sensitivity through increased expression and likely activity of several genes involved in the insulin signaling pathway in the liver and adipose tissues (Figure 8). Although simvastatin is able to lower cholesterol levels (Table 4), its effects on insulin signaling genes (Figures 5, 6, and 7) tended towards insulin resistance, in agreement with previous reports. Liver and adipose tissues are involved in development of insulin resistance, and in fact they have been proposed to be the organs from where the problem is initiated. Therefore, the enhanced sensitivity of insulin in these tissues suggests that EBN is effective at preventing insulin resistance. Furthermore, we hypothesize that synergism of multiple bioactives in EBN is contributing to the overall bioactivity observed.

## 4. Conclusions

In this study, we demonstrated that HFD will induce insulin resistance (higher OGTT, leptin and F2-isoprostane, and lower adiponectin levels), partly through transcriptional modulation of insulin signaling genes. Additionally, simvastatin was shown to further promote insulin resistance. EBN however is able to prevent insulin resistance by preventing some of the transcriptional changes on insulin signaling genes induced by HFD. There is need to further evaluate the potential use of EBN in the management of insulin resistance in already established insulin-resistant conditions.

## Figures and Tables

**Table 1:** Food composition and animal groups.

Animal group	Normal pellet	Cholesterol/cholic acid	Palm oil	Starch	Others
Normal	100%				
High fat diet	65%	5	20	10	
High fat diet + simvastatin	65%	5	20	10	Simvastatin (10 mg/kg)
High fat diet + 20% EBN	45%	5	20	10	20% EBN
High fat diet + 2.5% EBN	62.5%	5	20	10	2.5% EBN

EBN: edible bird's nest.

**Table 2:** Names, accession number, and primer sequences used in the study.

	Accession number	Left sequence	Right sequence
Irs2	NM_001168633	AGGTGACACTATAGAATAAGGCACTGGAGCCTTAC	GTACGACTCACTATAGGGAGCAGCACTTTACTCTTTCAC
Kcnj11	NM_031358	AGGTGACACTATAGAATACTACTTCAGGCAAAACTCTG	GTACGACTCACTATAGGGAGAACTTTCCAATATTTCTTTT
Insr	NM_017071	AGGTGACACTATAGAATAAGCTGGAGGAGTCTTCAT	GTACGACTCACTATAGGGAAAGGGATCTTCGCTTT
Gck	NM_001270849	AGGTGACACTATAGAATAATCTTTTGCAACACTCAGC	GTACGACTCACTATAGGGATTGTTGGTGCCCAGA
Pklr	NM_012624	AGGTGACACTATAGAATATCGGAGGTGGAAATTG	GTACGACTCACTATAGGGACTCTGGGCCGATTTT
Prkcd	NM_133307	AGGTGACACTATAGAATATCAAGAACCACGAGTTCA	GTACGACTCACTATAGGGATCTTTCTGGAAGATGGTG
B2m^∗^	NM_012512	AGGTGACACTATAGAATAATGCTTGCAGAGTTAAACA	GTACGACTCACTATAGGGATGCATAAAATATTTAAGGTAAGA
Hprt1^∗,#^	NM_012583	AGGTGACACTATAGAATATCCTCATGGACTGATTATG	GTACGACTCACTATAGGGACTGGTCATTACAGTAGCTCTT
Mapk1	NM_053842	AGGTGACACTATAGAATACATTTTTGAAGAGACTGCTC	GTACGACTCACTATAGGGAAACTCTCTGGACTGAAGAAT
Prkcz	NM_022507	AGGTGACACTATAGAATACTTTAACAGGAGAGCGTACT	GTACGACTCACTATAGGGATATTGTCATGTTTCCGAGAT
Ikbkb	NM_053355	AGGTGACACTATAGAATACTTGAACTTAAAGCTGGTTC	GTACGACTCACTATAGGGAACATTTTACTGTTGTCAAAGAG
Kan(r)^∗∗^			
Mtor	NM_019906	AGGTGACACTATAGAATATGGAACTTCGAGAGATGAG	GTACGACTCACTATAGGGATCACTTCAAACTCCACATAC
Actb^∗^	NM_031144	AGGTGACACTATAGAATAAACTACATTCAATTCCATCA	GTACGACTCACTATAGGGATAAAACGCAGCTCAGTAAC
Pik3r1	NM_013005	AGGTGACACTATAGAATACATCAGTATTGGCTTACG	GTACGACTCACTATAGGGATCATTTACTTCTTCCCTTGA

^∗^Housekeeping genes. ^#^Normalization gene. Underlined sequences are left and right universal left and right sequences (tags). ^∗∗^Internal control supplied by Beckman Coulter Inc (Miami, FL, USA) as part of the GeXP kit. RT conditions were 48°C for 1 min; 37°C for 5 min; 42°C for 60 min; 95°C for 5 min and then hold at 4°C. PCR conditions were initial denaturation at 95°C for 10 min, followed by two-step cycles of 94°C for 30 sec and 55°C for 30 sec, ending in a single extension cycle of 68°C for 1 min.

**Table 3:** Proximate analyses and lactoferrin, ovotransferrin, and sialic acid concentrations of edible bird's nest (EBN).

Bioactive/nutrient	EBN
Lactoferrin	4.68 ± 0.4 *μ*g/mg
Ovotransferrin	10.23 ± 0.8 *μ*g/mg
Sialic acid	110.4 ± 0.8 *μ*g/mg
Crude fat	0.54 ± 0.06%
Ash	4.0 ± 0.03%
Moisture	15.2 ± 0.02%
Carbohydrate	23.4 ± 0.29%
Crude protein	56.9 ± 0.27%

**Table 4:** Food intake and biochemical parameters.

Rat groups	Food intake		Chol. (mmol/L)	Trig. (mmol/L)	LDL (mmol/L)	HDL (mmol/L)	LDL/HDL	TG/HDL	Insulin (pg/mL)	HOMA-IR
	g/kg/day	Kcal/kg/day								
Normal	64.34 ± 10.96	215.54 ± 33.5^a^	1.55 ± 0.43^a^	0.62 ± 0.15^a^	0.28 ± 0.11^a^	1.18 ± 0.35^a^	0.24 ± 0.04^a^	0.55 ± 0.15^a^	495 ± 51.3^a^	1.91 ± 0.23^a,c,d^
Untreated control	48 ± 8.36	215.04 ± 37.45^a^	7.47 ± 1.13^b^	1.21 ± 0.38^b^	4.98 ± 1.03^b^	1.05 ± 0.13^a^	4.77 ± 0.98^b^	1.16 ± 0.33^b^	513.3 ± 38.8^a^	2.46 ± 0.22^b^
SIM	48.14 ± 8.17	215.67 ± 36.60^a^	4.99 ± 1.11^c,d^	0.63 ± 0.18^a^	3.6 ± 1.1^b,c^	1.04 ± 0.17^a^	3.46 ± 0.94^b,c^	0.62 ± 0.22^a,b^	602.1 ± 145.7^a^	2.83 ± 0.79^b,c^
2.5% EBN	48.23 ± 8.21	216.07 ± 36.78^a^	6.04 ± 0.75^b,c^	0.54 ± 0.1^a^	4.52 ± 0.71^b,c^	1.17 ± 0.18^a^	3.94 ± 0.88^b,c^	0.46 ± 0.08^a^	414.5 ± 18.8^b,c^	1.74 ± 0.09^c,d^
20% EBN	48.33 ± 8.00	216.52 ± 35.84^a^	4.17 ± 1.06^d^	0.44 ± 0.1^a^	2.98 ± 0.83^c^	1.18 ± 0.29^a^	2.63 ± 0.87^c^	0.38 ± 0.08^a^	426.7 ± 160.7^a.c^	1.63 ± 0.71^a,b,c^

Data represent mean ± SD (*n* = 6). Different alphabet in each column denotes significant difference (*P* < 0.05) in Tukey's multiple comparison test. Groupings are the same as Figure 2. HDL: high-density lipoprotein; HOMA-IR: homeostatic model assessment of insulin resistance; LDL: low-density lipoprotein; Chol.: cholesterol; SIM: Simvastatin; Trig.: triacylglyceride.

## Abbreviations

EBN: Edible bird's nest
Gck: Glucokinase
HFD: High fat diet
HOMA-IR: Homeostatic model assessment of insulin resistance
Ikbkb: Inhibitor of kappa light polypeptide gene enhancer in B-cells, kinase beta
Insr: Insulin receptor
IRS: Insulin receptor substrate
KCNJ11: Potassium inwardly rectifying channel, subfamily J, member 11
Mtor: Mammalian target of rapamycin
MAPK: Mitogen-activated protein kinase
OGTT: Oral glucose tolerance test
PI3K: Phosphoinositide-3-kinase
Pk: Pyruvate kinase
Prkcz: Protein kinase C, zeta.
